# Chemistry of Food Processing: Acrylamide in (Ultra-Processed) Food—A RASFF-Based Assessment

**DOI:** 10.3390/toxics14090738

**Published:** 2026-08-22

**Authors:** Đorđe Kitić, Branislava Srđenović Čonić, Ljilja Torović

**Affiliations:** 1 Department of Pharmacy, Faculty of Medicine, University of Novi Sad, Hajduk Veljkova 3, 21000 Novi Sad, Serbia; djordje_kitic@yahoo.com (Đ.K.); branislava.srdjenovic-conic@mf.uns.ac.rs (B.S.Č.); 2 Center for Medical and Pharmaceutical Investigations, Faculty of Medicine, University of Novi Sad, Hajduk Veljkova 3, 21000 Novi Sad, Serbia

**Keywords:** food processing, food safety, nova classification, public health

## Abstract

Chemical hazards generated during food production and processing represent a major food safety concern because of their toxic and carcinogenic potential. This study investigated the occurrence and distribution of acrylamide reported through the EU Rapid Alert System for Food and Feed (RASFF) alongside research data to evaluate temporal trends, food categories at risk, and the implications of current regulatory and mitigation measures. A total of 98 notifications were identified between 2009 and 2025, with a marked increase following the implementation of EU regulatory measures in 2017 and 2019. However, consistently high numbers of notifications were observed only during the final three years of the study period. Acrylamide was predominantly associated with thermally processed foods, particularly cereals and bakery products, and prepared dishes and snacks. The geographical distribution of notifications partially reflected established production and trade patterns, with Bosnia and Herzegovina, Serbia, Ukraine, and the Netherlands representing the most frequently reported countries of origin. Classification of the reported products according to the NOVA system, combined with RASFF risk decisions and notification classifications, revealed a marked concentration of high-risk notifications in ultra-processed foods. These products accounted for 85.7% of notifications classified as posing a serious risk and 77.3% of alert notifications, underscoring their disproportionate contribution to acrylamide-related food safety concerns. Overall, the findings demonstrate that, despite the implementation of regulatory measures and mitigation strategies, acrylamide remains a persistent challenge throughout the food chain, highlighting the need for continuous monitoring, enhanced analytical surveillance, and more effective mitigation approaches.

## 1. Introduction

In the modern era of food production, hazardous chemicals constitute a major food safety concern due to their potential adverse effects on human health. Contamination may arise from polluted air, soil, or water, as well as from inadequate transport, storage, or pre-treatment conditions. In particular, chemical contaminants generated during food processing have become increasingly prevalent in widely consumed mass-produced foods [1,2].

To ensure the availability of safe, palatable, and nutritionally adequate food for a continuously growing global population, food processing has become an essential component of modern food systems. Depending on the type of energy applied, food processing operations may be classified as thermal, mechanical, radiation-based, chemical, or biochemical processes [3]. The transformation of raw materials into finished food products, as well as microbiological safety and extended shelf life, often requires the application of one or more of these processing methods.

Nevertheless, food processing has also raised substantial public health concerns in both industrial food production and domestic cooking practices. Many foods are subjected to elevated temperatures to improve safety, enhance sensory properties, prolong shelf life, or facilitate preparation, particularly for baked and fried products. However, exposure to high temperatures may also result in the formation of a wide array of toxic compounds associated with adverse health outcomes exerted after chronic dietary exposure [4,5].

Acrylamide is formed during the thermal processing of carbohydrate-rich foods through the Maillard reaction, which is responsible for the characteristic flavor, color, and texture of baked and fried products. During this process, reaction intermediates known as Schiff bases are generated and subsequently undergo a series of complex chemical transformations that ultimately lead to acrylamide formation. Under conditions of high temperature and low moisture content, free reducing sugars react with free amino acids, particularly asparagine, resulting in the formation of acrylamide [6,7].

Following its discovery in food products in 2002, the European Food Safety Authority (EFSA) was tasked with evaluating the potential health risks associated with dietary exposure to acrylamide. Acrylamide and its metabolites are considered genotoxic and carcinogenic, as even low levels of exposure to genotoxic compounds may induce DNA damage. Consequently, the EFSA was unable to establish a tolerable daily intake for acrylamide. Instead, risk assessment was based on benchmark dose modeling, defining exposure levels associated with an increased probability of adverse effects, including neoplastic and neurotoxic outcomes. The lower confidence limit of the benchmark dose associated with a 10% increase in response (BMDL_10_) was identified as 0.17 mg/kg bw/day for tumor formation and 0.43 mg/kg bw/day for neurological effects [8]. In 2022, the EFSA revisited the evidence for genotoxicity of acrylamide and confirmed the margin of exposure (MOE) approach for risk characterization of its neoplastic effects, as recommended for substances that are both genotoxic and carcinogenic [9]. The International Agency for Research on Cancer (IARC) has classified acrylamide as a probable human carcinogen (Group 2A) [10], emphasizing the need for strict regulatory control.

Currently, Regulation (EU) 2017/2158 is mandatory for all food business operators within the European Union (EU), establishing benchmark levels for acrylamide in various food categories, along with required mitigation measures, monitoring procedures, and documentation practices. Furthermore, Recommendation (EU) 2019/1888 expanded the range of food categories subject to acrylamide monitoring [9,11,12].

Given the established health risks associated with acrylamide exposure and the unavoidable thermal processing of many food products, extensive efforts have focused on developing reliable detection and mitigation strategies. Currently, gas chromatography–mass spectrometry (GC–MS) and liquid chromatography–mass spectrometry (LC–MS) are considered the reference analytical methods for acrylamide detection. However, their relatively low throughput, complex sample-preparation requirements, and operational costs have spurred interest in alternative approaches, particularly biosensor-based technologies. These methods are generally based on electrochemical or biorecognition systems designed for rapid acrylamide detection in food matrices [13,14].

In response to rising public health concerns, the declining overall quality of modern dietary patterns, and the need to establish contemporary nutritional guidelines, several food classification systems have been developed to categorize foods by degree of processing. Frameworks such as the International Food Information Council system, the National Institute of Public Health in Mexico classification, the International Agency for Research on Cancer–European Prospective Investigation into Cancer and Nutrition (EPIC) classification, the Poti system, and the Siga system were designed to investigate epidemiological associations between industrially processed foods and nutritional health outcomes. Among these systems, the NOVA classification has emerged as the most widely recognized global framework, categorizing foods into four groups according to the nature, extent, and purpose of industrial processing: unprocessed or minimally processed foods (Group 1), processed culinary ingredients (Group 2), processed foods (Group 3), and ultra-processed foods (Group 4) [3,15].

However, these classification systems are primarily based on physical formulation and the presence of added ingredients, without accounting for the formation of neoformed contaminants (NFCs) generated during thermal processing. For example, products such as roasted coffee and skimmed milk powder are classified within the NOVA framework as minimally processed foods (Group 1), despite the fact that high-temperature roasting and spray-drying processes may promote the formation of hazardous compounds, including acrylamide, furans, and 5-hydroxymethylfurfural (HMF). Consequently, reliance solely on processing-based nomenclature may provide an incomplete assessment of chemical food safety and toxicological risk. This limitation highlights the necessity for independent regulatory frameworks based on direct toxicological evaluation and contaminant monitoring, rather than exclusively on the intended degree of industrial processing [15,16].

The EU legislative framework is widely regarded as one of the most comprehensive and stringent food safety systems worldwide. As part of its integrated food safety infrastructure, the Rapid Alert System for Food and Feed (RASFF) was established to facilitate the rapid exchange of information among member states and to enable timely risk management actions by food safety authorities in situations involving potential threats to public health within the food chain [4]. The legal basis for this system is Article 50 of Regulation (EC) No 178/2002, commonly referred to as the General Food Law [16]. Access to the RASFF network is restricted to the competent authorities of member states and the European Commission. However, a publicly accessible interactive online platform, known as the RASFF Window, provides summary information on recently transmitted notifications and enables retrospective searches of historical notifications [16].

The present study aimed to provide a comprehensive overview of acrylamide in food, identify potential patterns in its occurrence and distribution, improve understanding of its origin, and evaluate the effectiveness of current detection, monitoring, and mitigation strategies.

## 2. Materials and Methods

A targeted search of the Rapid Alert System for Food and Feed (RASFF) database was conducted on 20 th January 2026 to identify notifications classified as “food” (chosen within Notification types in the Type search filter) and related to acrylamide, reported between 01.01.2009 and 31.12.2025 (the initial screening showed no records related to acrylamide before 2009). The search was performed with the Subject search filter using the term “acrylamide”.

The retrieved data were exported to Microsoft Excel (Version 2607 (Build 20228.20190 Click-to-Run)) (Microsoft Corporation, Redmond, WA, USA) [17] for processing and analysis. The resulting dataset was analysed by food category, hazard type, risk decision, notification classification, and country of origin (all parameters defined as in the RASFF). These variables were subsequently grouped, cross-tabulated, and evaluated to identify patterns and associations. Minor discrepancies in category totals may occur due to incomplete notification records that could not be unequivocally assigned to a specific variable category.

The study employed a retrospective descriptive design, and the findings were expressed as absolute numbers and percentages.

The geographical distribution of notifications was visualized using the map function in Microsoft Excel (Version 2607 (Build 20228.20190 Click-to-Run)), with embedded the Bing Maps service [17] and integrated geospatial information from OpenStreetMap [18] and GeoNames [19] is embedded in Microsoft Excel Version 2607 (Build 20228.20190 Click-to-Run).

To support risk prioritization, the relationship between acrylamide risks and food processing level was investigated using a risk matrix with low–medium–high–severe gradation of both variables. Products described in the RASFF subject field were classified according to the NOVA food classification system: each product was assigned to one of four classes based on its degree of processing: Class 1 (unprocessed or minimally processed foods), Class 2 (processed culinary ingredients), Class 3 (processed foods), and Group 4 (ultra-processed foods). The RASFF data were subsequently integrated with the NOVA classification to construct two risk matrices illustrating the distribution of notifications across food processing classes according to (1) RASFF risk decisions and (2) notification classifications.

RASFF risk decisions indicate the outcome of the risk assessment performed by the notifying authority and classify notifications as serious risk, potentially serious risk, potential risk, no risk, undecided, or not assessed, depending on whether adverse health effects can be proven, suspected, or ruled out based on the available scientific evidence and human exposure levels. Risk decisions definitions as assigned in the RASFF are briefly summarized: Serious risk: a direct or indirect threat to human health that requires rapid, urgent intervention; Potentially serious risk: analytical results or contamination levels suggest a probable health hazard, though it may lack complete final verification; Potential risk: a definitive serious risk is not clearly suspected, but an adverse impact cannot be entirely ruled out (incomplete or insufficient data); No risk: a health risk can be completely ruled out; Undecided: final toxicological or exposure data is still under review (an inquiry or exchange was initiated); Not assessed: a formal evaluation of the hazard’s severity has not yet been performed [16].

RASFF notification classifications describe urgency of the reported event: alert notifications, issued when a food presenting a serious risk is already on the market and rapid action is required; information notifications, used when a risk has been identified but immediate action by other members is not required because the product is not on the market or the risk is otherwise under control; and border rejection notifications, concerning consignments rejected at the EU border due to identified food safety risks [16].

The RASFF data were interpreted alongside market surveillance studies including comparisons before and after the implementation of mitigation measures, from various countries. These studies were assessed to explore evidence on long-term trends and the effectiveness of preventive strategies aimed at reducing acrylamide formation.

## 3. Results and Discussion

### 3.1. Acrylamide Notification Trend by Years

**Figure 1:**
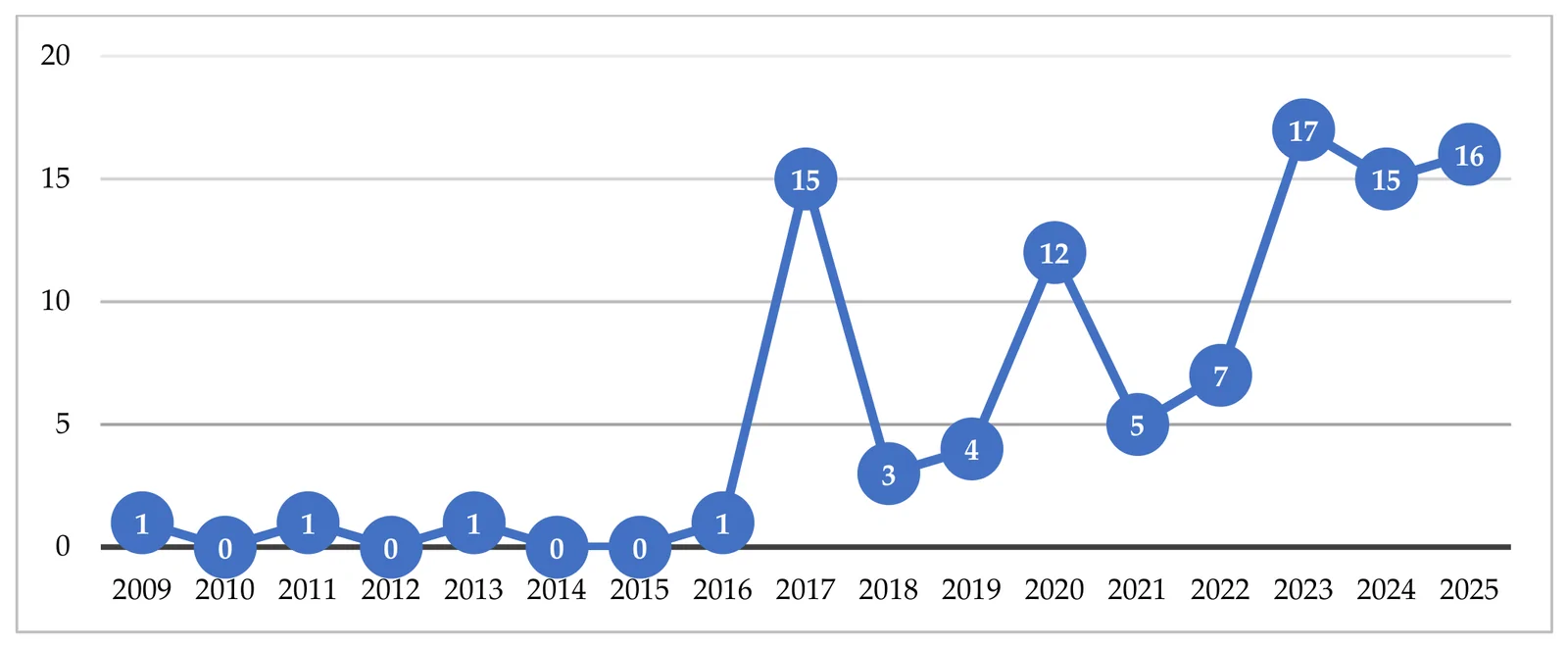
Temporal distribution of acrylamide notifications (RASFF, 2009–2025).

A total of 98 notifications related to acrylamide were identified between 2009 and 2025. Figure 1 illustrates the temporal distribution of these notifications.

Although acrylamide was first identified in food products in 2002, specific regulatory measures were not introduced until 2017, followed by updated recommendations and enhanced monitoring guidelines in 2019, which substantially expanded the range of food products subject to surveillance [11,12]. The pronounced increase in acrylamide-related notifications observed after these regulatory milestones—the rise associated with the 2019 revisions became evident in the 2020 data—is therefore expected, but likely reflects intensified monitoring and enforcement rather than a true increase in contamination. More noteworthy, however, is the consistently high number of notifications recorded during the final three years of the study period, suggesting that AA remains a persistent regulatory and food safety challenge despite the implementation of mitigation measures.

Acrylamide time/market trends are discussed in Section 3.7.

### 3.2. Acrylamide Notification Trend by Product Matrices

**Figure 2:**
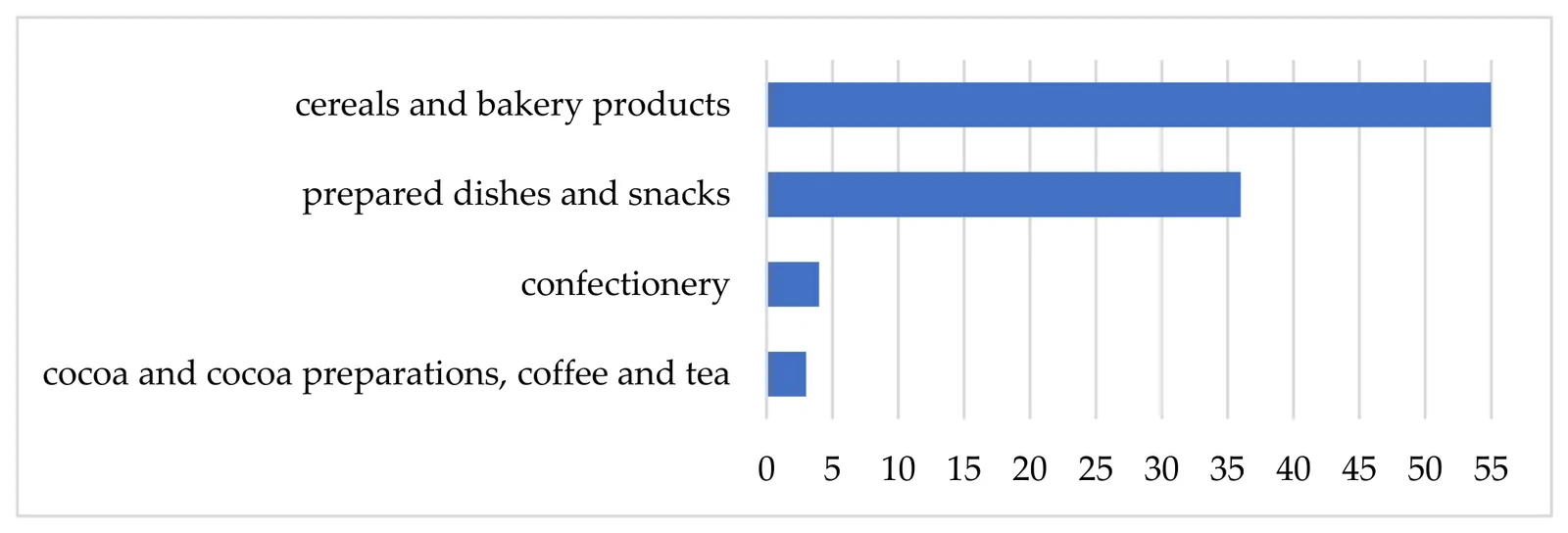
Distribution of acrylamide notifications across food categories (RASFF, 2009–2025).

Potential associations between acrylamide and food categories (Figure 2) were analysed to identify possible causative relationships.

Acrylamide was most frequently detected in cereals and bakery products (mainly biscuits), and prepared dishes and snacks (mainly vegetable (potato) chips). These food categories are subjected to thermal processing methods such as baking, frying, or roasting. In addition, they contain considerable amounts of starch, reducing sugars, and the amino acid asparagine. In combination with elevated temperatures and low moisture conditions, these factors create optimal conditions for acrylamide formation through the Maillard reaction [8].

**Figure 3:**
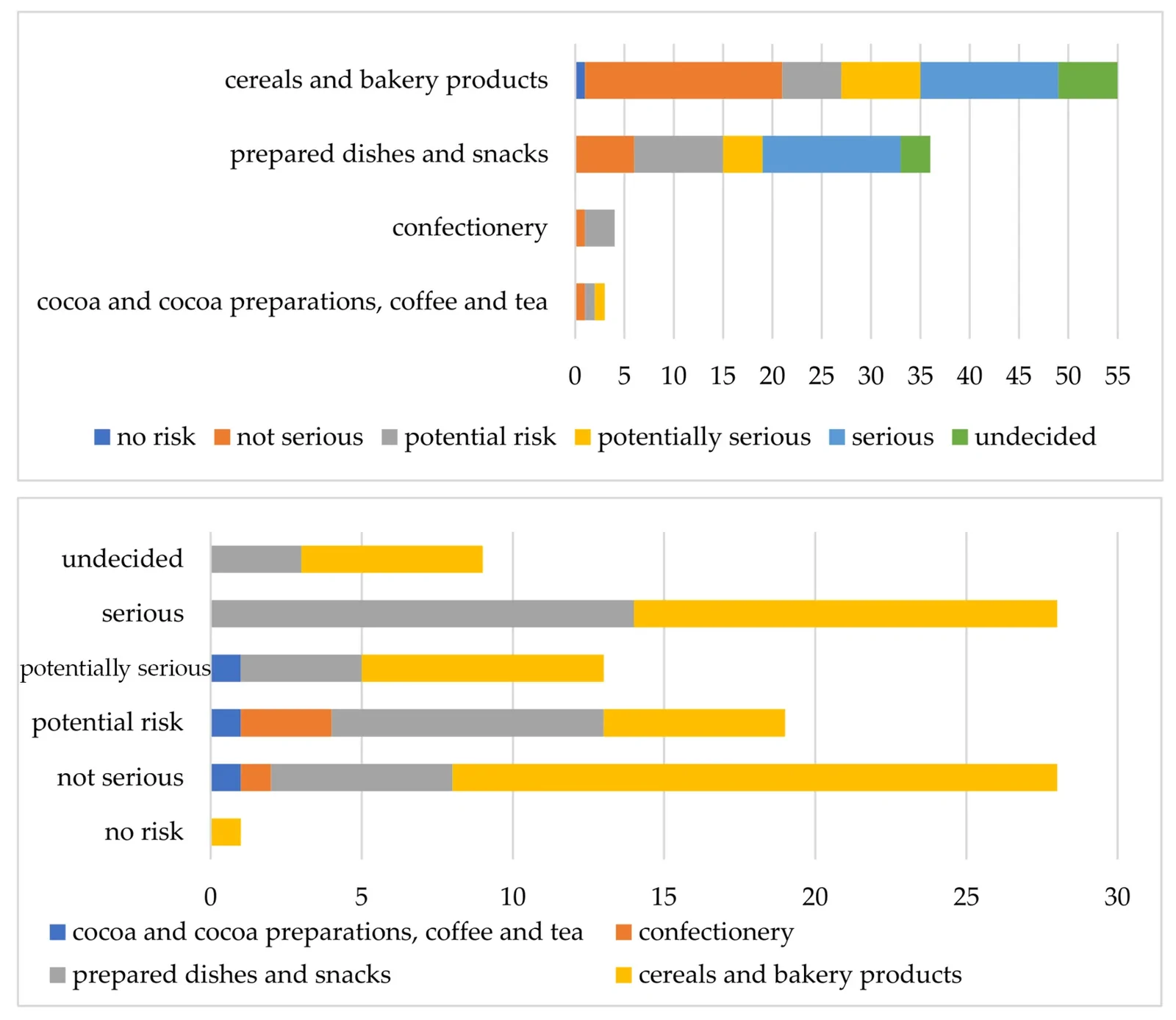
Distribution of acrylamide notifications by risk decision (RASFF, 2009–2025).

A total of 28 (28.6%) acrylamide-related notifications were categorized as serious risk, equally involving cereals and bakery products, and prepared dishes and snacks (Figure 3). The considerable proportion of notifications classified as “undecided” reflects the regulatory context, indicating that a definitive risk assessment could not always be reached at the time of reporting. In many cases, acrylamide presence in foodstuffs was evident, yet the available scientific evidence was insufficient to support a robust evaluation of risk severity within the RASFF framework. Such uncertainty also poses practical challenges for risk management, as competent authorities must determine appropriate enforcement measures despite the absence of a clear risk classification. Conversely, the substantial proportion of notifications classified as “serious risk” demonstrates that many cases were considered sufficiently significant to warrant immediate regulatory intervention and rapid implementation of control measures.

**Figure 4:**
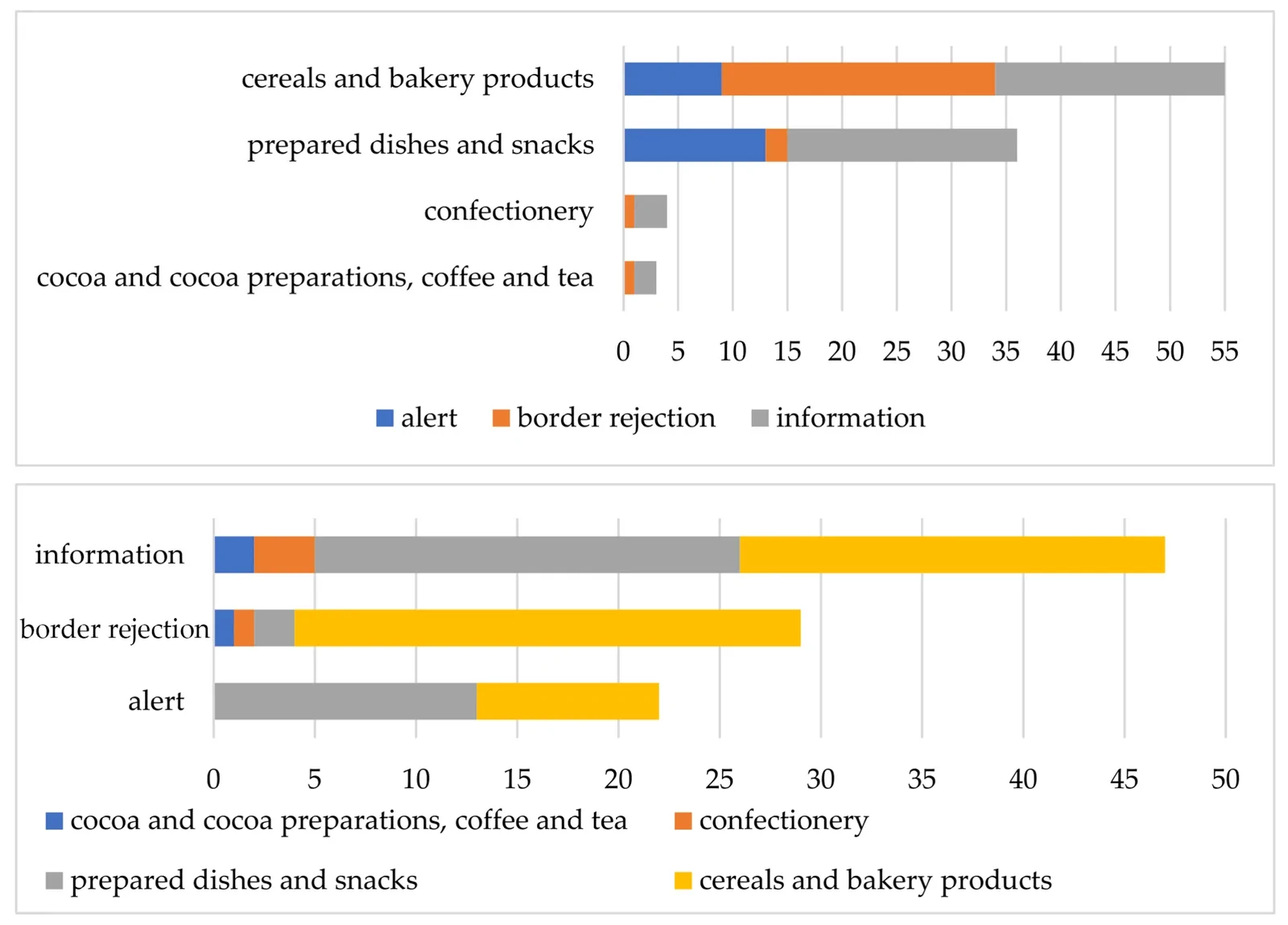
Distribution of acrylamide notifications by notification classification (RASFF, 2009–2025).

The majority of notifications were classified as information (42 cases; 89.4%) (Figure 4), equally attributed to cereals and bakery products, and prepared dishes and snacks.

The predominance of information notifications can be attributed to the fact that they primarily serve to facilitate information exchange and support follow-up actions, even when an immediate health risk has not been formally established. Nevertheless, the considerable proportion of alert notifications (22.4%) indicates that many notified products had already been placed on the market, necessitating rapid intervention by the competent authorities to prevent or limit consumer exposure.

### 3.3. Acrylamide Notification by Product Origin

**Figure 5:**
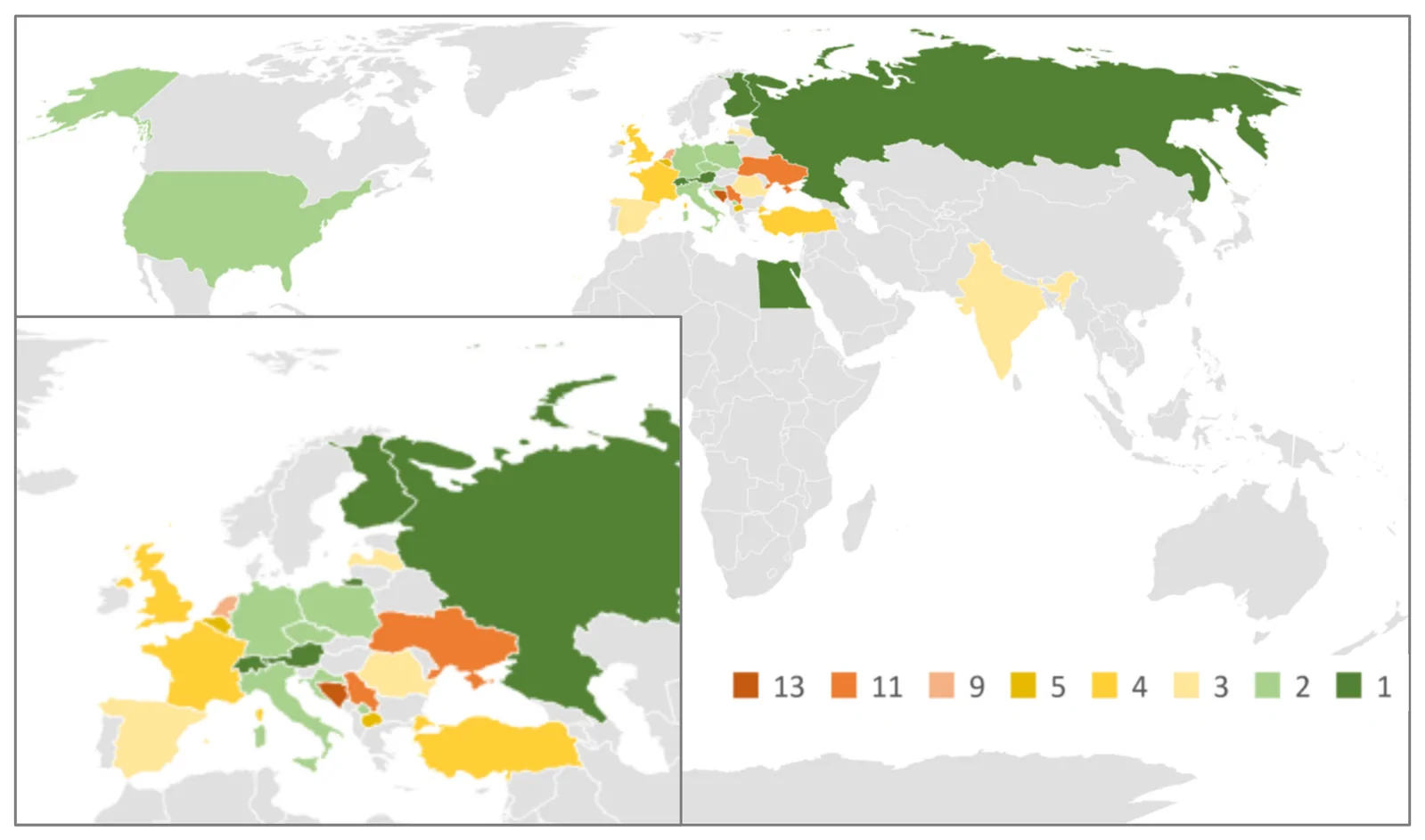
Geographic distribution of acrylamide notifications—origin countries (RASFF, 2009–2025) (Map generated using Microsoft^®^ Excel^®^ Powered by Bing™. Contains data from GeoNames, Microsoft, Open Places, and OpenStreetMap).

Acrylamide-related notifications were analysed according to country of origin and subsequently mapped (Figure 5). The countries associated with the highest numbers of reported cases were Bosnia and Herzegovina (13 notifications), Serbia and Ukraine (13 each), and the Netherlands (9).

However, the geographical distribution of RASFF notifications according to country of origin only partially reflects established global production and trade networks. Ukraine is among the world’s leading exporters of wheat, flour, and other starch-rich commodities. As previously discussed, such products are commonly associated with acrylamide, particularly following thermal processing. Bosnia and Herzegovina and Serbia are European countries but are not EU Member States, which may influence the implementation and effectiveness of EU acrylamide mitigation measures. Market surveillance of commercially available corn-based snack products in Serbia revealed substantial variability in acrylamide concentrations, with approximately 37% of analysed retail snack products exceeding the EU benchmark levels. These findings demonstrate a persistent discrepancy between regulatory standards and the actual chemical safety status of processed snack products available on regional markets [20]. In the case of the Netherlands, the high number of notifications may partly reflect its role as a major European trade and distribution hub rather than contamination originating exclusively from domestic production. The widespread international consumption of industrially processed food products contributes to extensive exposure patterns that are not confined to manufacturing regions but instead extend throughout importing countries.

The role of notifying countries in the RASFF system should also be considered when interpreting the data. However, their contribution primarily reflects differences in official control activities rather than the actual distribution of non-compliances. Notification patterns are influenced by variations in surveillance intensity, analytical capacity, regulatory priorities, and the strategic role of individual countries as major entry points or distribution hubs within the EU food supply chain.

### 3.4. Risk Prioritization—Mapping of the RASFF Findings to the NOVA Food Classification

Classification of RASFF-reported food products according to the NOVA system revealed a clear predominance of ultra-processed foods, accounting for 84.7% of all acrylamide-related notifications. In contrast, minimally processed foods (NOVA class 1) and processed culinary ingredients (NOVA class 2) were only sporadically represented, while processed foods (NOVA class 3) contributed a relatively small proportion of notifications.

**Figure 6:**
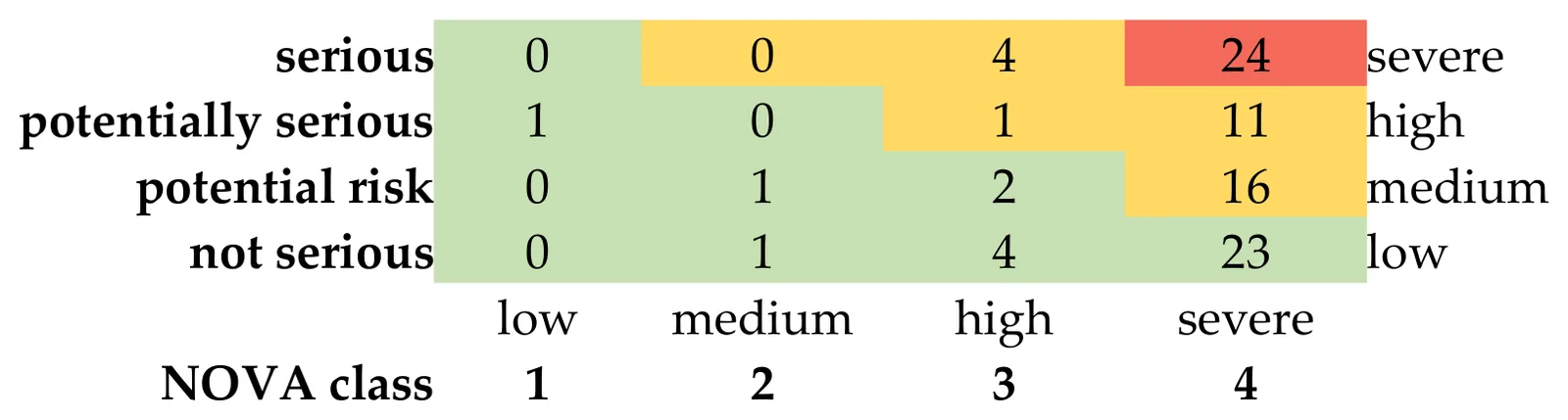
Risk-matrix: RASFF risk decision (ordered by degree of risk severity, from not serious–low to serious–severe)—NOVA food classes (ordered by degree of food processing, from 1–low to 4–severe). Color legend: high (red), medium (yellow), and low (green) priorities. Note: notifications categorized as “undecided” were omitted from the risk matrix.

**Figure 7:**
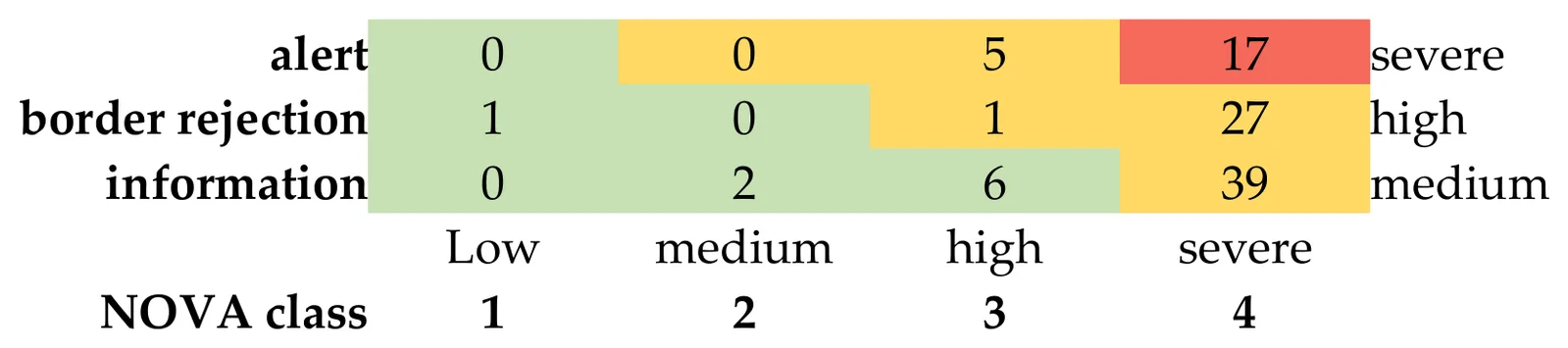
Risk-matrix: RASFF notification classification (ordered by degree of severity, from information–medium to alert–severe)—NOVA food classes (ordered by degree of food processing, from 1–low to 4–severe). Color legend: high (red), medium (yellow), and low (green) priorities.

Risk matrices integrating the NOVA classification with the RASFF risk decision and notification classification variables demonstrated that ultra-processed foods accounted for 85.7% of products classified as posing a serious risk due to acrylamide (Figure 6) and 77.3% of products associated with alert notifications (Figure 7), underscoring their disproportionate contribution to the most critical acrylamide-related food safety concerns.

Figure 6 demonstrates a clear association between the degree of food processing and the severity of RASFF risk decisions. Ultra-processed foods (NOVA class 4) accounted for the vast majority of notifications across all risk categories. Within the ultra-processed food group, 32.4% of notifications were classified as posing a serious risk, whereas 14.9% and 21.6% were classified as posing a potentially serious risk and a potential risk, respectively.

Figure 7 shows that within the ultra-processed foods group, 20.5% were classified as alerts, 32.5% as border rejections, and the remaining 47.0% as information notifications.

These findings emphasize the importance of prioritizing mitigation strategies and regulatory surveillance for ultra-processed foods, reflecting their susceptibility to acrylamide formation during industrial processing.

### 3.5. Literature Synthesis of Acrylamide-Content Variance

The RASFF data were interpreted alongside published market surveillance studies from various countries. An evaluation of the worldwide occurrence of acrylamide in foods through a systematic review, meta-analysis, and meta-regression included 47 studies (230 datasets) published up to 2019. The authors compared acrylamide concentrations across major food categories and countries, and investigated whether socioeconomic indicators are associated with acrylamide contamination. The analysis showed that potato-based products contained the highest pooled acrylamide concentrations (740 µg/kg), followed by fried foods, breakfast cereals, coffee, chocolate, and infant foods, whereas nuts contained the lowest levels. The study also highlights considerable variability among countries, reflecting differences in raw materials, food processing practices, and dietary habits. A novel aspect of the study is the meta-regression analysis, which demonstrated a significant association between socioeconomic status and acrylamide contamination: countries with lower Gross Domestic Product (GDP) and the Human Development Index (HDI) rankings tended to report higher acrylamide concentrations, suggesting that differences in food quality control, processing technologies, and regulatory implementation may influence contamination levels [21].

The most recent market surveillance studies (Table 1) demonstrate that acrylamide remains a persistent contaminant in thermally processed foods. Although many products comply with current EU benchmark levels, substantial variability persists both within and between food categories, reflecting differences in raw materials, processing conditions, and the implementation of mitigation strategies.

Potato-based products continue to represent one of the greatest challenges, with several investigations reporting average concentrations exceeding EU benchmark levels and a considerable proportion of non-compliant samples [22,23,24,25,26]. Elevated acrylamide concentrations were also frequently observed in breakfast cereals [28], biscuits [31,32], wafers [31], popcorn [34], and roasted coffee [36], confirming that intensively heat-processed carbohydrate-rich foods remain major contributors to dietary acrylamide exposure.

At the same time, recent investigations demonstrate that effective mitigation is achievable. Compliant acrylamide concentrations have been reported for potato crisps [26], ready-to-eat snacks [27], crackers [26], coffee [26], and cafeteria meals [33,37], indicating that optimized processing conditions, careful raw material selection, and targeted technological interventions can substantially reduce acrylamide formation. Nevertheless, the pronounced variability among studies suggests that the implementation of mitigation strategies remains inconsistent across the food industry, highlighting the continuing challenge of reducing acrylamide while maintaining desirable sensory quality.

Among studies evaluating the content of contaminants in food, Total Diet Studies (TDS) deserve particular attention because they provide a comprehensive assessment of dietary exposure under real-world consumption conditions. TDS generates representative data on the average concentrations of chemical substances in foods and constitutes a cornerstone of chronic dietary exposure assessment. Their design reflects the dietary habits of the target population by analysing foods as they are commonly consumed, after preparation using typical household cooking practices, and by grouping similar food items [38]. This approach captures changes in food composition arising during preparation, including both the degradation of existing compounds and the formation of process-induced contaminants. The first German Total Diet Study (BfR MEAL) analyzed 230 foods and demonstrated that thermal processing in both industrial production systems and household cooking environments generates considerable baseline levels of acrylamide across frequently consumed food products. Particularly elevated acrylamide concentrations were identified in vegetable crisps (1430 μg/kg), potato pancakes (558 μg/kg), and pan-fried potato products (450 μg/kg). Furthermore, the study reported that intensive browning conditions, such as highly browned French fries, consistently exceeded EU benchmark levels regardless of the cooking method applied. Household preparation methods significantly impact acrylamide reduction, with oven-baking for French fries and air-frying for sweet potato fries resulting in the lowest levels [38]. These findings indicate that even within highly regulated food systems, localized processing practices may substantially influence contaminant formation alongside broader trade-related factors.

### 3.6. Acrylamide Mitigation Strategies

Mitigation strategies for acrylamide reduction require a multi-level “farm-to-fork” approach, which can generally be divided into pre-processing and processing approaches. Pre-processing measures include the selection of low-precursor potato cultivars (lower concentrations of free asparagine and reducing sugars), and utilization of CRISPR/Cas9 technology to edit asparagine biosynthesis genes, as well as optimization of storage conditions [39]. Processing-related strategies primarily involve controlling technological parameters such as temperature, pressure, and processing duration. Many food manufacturers currently use lower processing temperatures, typically below 200 °C, combined with optimized processing times to minimize AA formation while maintaining acceptable sensory and organoleptic properties [15]. Emerging technologies, including ultrasound treatment and gamma irradiation, are also being explored as potential mitigation tools, although additional studies are necessary to evaluate their industrial feasibility, economic viability, and scalability [13,14,40].

Additional mitigation strategies include the use of probiotic microorganisms, antioxidants, and enzymes such as asparaginase. These approaches may reduce acrylamide formation by inhibiting key reaction pathways or decreasing the availability of reaction precursors. While certain *Lactobacillus* strains have shown potential for reducing acrylamide levels, their effectiveness under industrial processing conditions remains limited and requires further investigation [40]. Despite their promise, current evidence suggests that many of these emerging technologies remain insufficiently validated for large-scale industrial application or demonstrate limited effectiveness under real processing conditions.

The use of exogenous additives—including amino acids, organic acids, metal ions, and natural antioxidants—can reduce acrylamide formation by interfering with the Maillard reaction or competing with key precursors, particularly asparagine. Among these approaches, asparaginase treatment has emerged as the most advanced and commercially feasible strategy, especially in bakery products, where acrylamide reductions of up to 85% have been reported without compromising sensory quality. However, this approach involves important trade-offs. Although additives such as glycine, cysteine, and various metal salts can markedly inhibit acrylamide formation, they may also promote the formation of secondary toxic by-products, including 5-HMF, furans, and heterocyclic aromatic amines. Moreover, additive efficacy is strongly matrix-dependent and concentration-sensitive. Certain natural antioxidants and metal ions may, under specific conditions, inadvertently enhance acrylamide formation or cause undesirable sensory changes, such as bitterness or sourness, if not carefully optimized [41].

Ingredient selection presents a critical challenge in acrylamide mitigation, particularly when nutritional enhancement is prioritized. A study on biscuit reformulation demonstrated that wholegrain and alternative flours, including rye, oat, and spelt, provide important nutritional benefits through increased dietary fiber and protein content but also contain higher concentrations of acrylamide and hydroxymethylfurfural (HMF) precursors, particularly free asparagine and reducing sugars. Consequently, formulations based on wholegrain flours generally produce higher levels of these process contaminants than those prepared with refined wheat flour. Among the evaluated cereals, rye generated the highest acrylamide concentrations, whereas buckwheat emerged as a promising alternative owing to its naturally low free asparagine content. Maintaining an appropriate balance between nutritional quality and chemical safety, therefore, requires careful optimization of processing conditions. Standard baking conditions (180 °C for 10 min) generally maintained acrylamide concentrations below current EU benchmark levels, whereas more intensive thermal processing resulted in exceedances. These findings highlight the importance of integrating nutritional reformulation with precisely controlled processing parameters and targeted mitigation strategies to ensure that improvements in nutritional quality are not achieved at the expense of food safety [30].

Of particular relevance in populations with high rice consumption, rice and rice-based products generally contain lower acrylamide concentrations than other cereal products, primarily because of their lower content of free asparagine, the principal precursor of acrylamide formation. However, the cooking method remains the dominant determinant of acrylamide formation. Dry-heat processes, including frying, roasting, and puffing, markedly increase acrylamide concentrations, particularly in crispy products such as rice crackers and puffed cereals, whereas moist-heat methods, including boiling and steaming, produce negligible or undetectable levels. These characteristics make rice flour an attractive low-acrylamide alternative for industrial snack production, with reported reductions in acrylamide formation of up to 34.5% when substituted for conventional cereal flours. Furthermore, simple pre-treatment strategies, such as washing or soaking rice in water or citric acid, can reduce acrylamide formation by more than 90%. Collectively, these findings highlight the potential of combining ingredient selection with optimized processing practices to substantially reduce dietary acrylamide exposure, particularly in regions where rice constitutes a dietary staple [42].

Effective mitigation now spans a multidisciplinary framework, ranging from asparaginase treatment and innovative vacuum frying to the synergistic use of polyphenols and dietary fibers, which can reduce acrylamide formation and bioavailability by up to 64% [43]. Furthermore, consumer-level practices—such as avoiding refrigeration of raw potatoes to prevent “cold-induced sweetening” and frying to a light golden color—are vital for minimizing dietary exposure [39].

### 3.7. Acrylamide Time/Market Trends

To evaluate the long-term effectiveness of acrylamide mitigation strategies under real-world market conditions, particular attention should be given to studies assessing temporal trends in acrylamide concentrations in commercially available foods. Accordingly, the RASFF analysis was complemented by long-term market monitoring studies, including before-and-after comparisons following the implementation of mitigation measures, providing additional evidence on temporal trends and the effectiveness of preventive strategies.

Analysis of acrylamide concentrations in European potato crisps between 2002 and 2019 indicates that, although mean levels have largely plateaued since 2011, average concentrations have declined by 54% since acrylamide was first identified as a food contaminant. While further reductions in the overall mean appear increasingly difficult to achieve, the continued decline in the 85th, 90th, and 95th percentiles demonstrates that manufacturers have successfully reduced the occurrence of products with exceptionally high acrylamide concentrations. This improvement is further reflected in the proportion of market samples exceeding the EU benchmark level, which decreased from more than 40% in 2002 to 7.75% in 2019. The greatest progress was observed in Northern and Eastern Europe, where historically elevated acrylamide concentrations have gradually converged toward the lower levels typically reported in Southern and Western Europe. Nevertheless, seasonal variation remains a major challenge. Acrylamide concentrations consistently peak between November and May owing to the accumulation of reducing sugars during potato storage, limiting the ability of manufacturers to maintain compliance with benchmark levels throughout the year [44].

A comprehensive survey of potato products marketed in Poland between 2004 and 2020 demonstrated a statistically significant decline in acrylamide concentrations across all evaluated categories, including potato crisps, restaurant-prepared French fries, and French fries produced from frozen semi-finished products. Throughout the study period, potato crisps consistently exhibited the highest median acrylamide concentration (388 μg/kg), followed by restaurant-prepared French fries (272 μg/kg), whereas frozen semi-finished products contained the lowest median concentration (172 μg/kg). Over the 17 years, median acrylamide concentrations decreased nearly fourfold in frozen semi-finished products and by almost 50% in potato crisps. Furthermore, between 2011 and 2020, when indicative and subsequently benchmark levels were introduced, fewer than 2.5% of samples exceeded the applicable regulatory thresholds. These findings suggest that implementing mitigation strategies, including selecting low-sugar potato cultivars and optimizing thermal processing conditions, has substantially reduced acrylamide formation in commercial potato products [45].

A two-decade surveillance study of the Spanish market indicates a concerning reversal of the previously declining trend in acrylamide concentrations in potato crisps. Although average concentrations decreased by 55% between 2004 and 2019, the 2025 survey revealed that 51% of analyzed samples exceeded the EU benchmark level of 750 μg/kg, with the median concentration increasing to 785 μg/kg. This loss of sustained compliance has been attributed to the uneven implementation of mitigation strategies across manufacturers. In addition, the EU-wide ban on chlorpropham, formerly used as a potato sprout suppressant, has likely contributed to this trend by increasing the accumulation of reducing sugars during potato storage, thereby promoting acrylamide formation during frying. Collectively, these findings suggest that current reformulation and mitigation strategies for potato crisps have not fully adapted to evolving regulatory requirements and changes in raw material characteristics, highlighting the need for renewed surveillance and more harmonized industrial practices [23].

In contrast, the same two decades of product reformulation have resulted in a substantial reduction in acrylamide concentrations in Spanish breakfast cereals, with mean levels declining from 292 μg/kg in 2006 to 114 μg/kg in 2025. By 2025, 95% of products complied with EU benchmark levels, compared with 85% in 2018, demonstrating the effectiveness of industry-wide mitigation measures. These improvements coincided with a shift toward nutritionally improved formulations characterized by lower sugar and saturated fat contents and greater incorporation of whole grains, high-fiber ingredients, and alternative cereals such as oats, spelt, and rye. A paired analysis of brands marketed in both 2018 and 2025 further showed that store-brand cereals achieved a statistically significant 68.5% reduction in mean acrylamide concentrations, indicating proactive adoption of mitigation strategies by retailers in response to regulatory requirements. Although the incorporation of bran and other fiber-rich ingredients may increase the availability of acrylamide precursors, the successful application of mitigation measures, including asparaginase treatment and optimized thermal processing, has enabled manufacturers to enhance nutritional quality while maintaining chemical safety [29].

Recent evidence indicates that the implementation of the ALARA (“As Low As Reasonably Achievable”) principle and increased awareness among food manufacturers have contributed to a general decline in acrylamide concentrations in German foods since 2010. Nevertheless, relatively high concentrations continue to be detected in specific product categories, notably snack foods such as popcorn, salty sticks, and dark chocolate. These results emphasize the importance of avoiding over-browning foods—following the motto “golden instead of charred”—to effectively minimize dietary exposure [38].

Although the implementation of EU Regulation 2017/2158 is estimated to have reduced acrylamide levels by an average of 15–20% across regulated food categories, concentrations in certain products have remained stable or even increased slightly [46]. This persistence is largely driven by consumer demand for specific sensory characteristics that limit mitigation efforts. Coffee and cocoa products are notable examples, as darker roasting profiles favored for their flavor and aroma promote increased acrylamide formation [43]. Coffee is a significant source of dietary acrylamide for adults, with concentrations in dry powder typically ranging from 249 to 710 μg/kg. Robusta beans generally contain higher levels of acrylamide than Arabica because they possess a greater initial concentration of free asparagine, which is the primary precursor for its formation. Although acrylamide forms early in the roasting process via the Maillard reaction, its concentration peaks and then decreases significantly as roasting continues toward darker degrees, as the rate of thermal degradation eventually exceeds the rate of formation. Because the compound is highly water-soluble, it is extracted into the final brew at a very high rate of 92% to 99% [47].

### 3.8. Study Limitations

Concerning study limitations, it has to be noted that in analyses based on the RASFF data, the frequency of notifications does not necessarily reflect the true prevalence of a contaminant. Temporal increases in notifications may instead result from changes in regulatory priorities, the expansion of monitoring programs, improved analytical capabilities, or enhanced reporting practices, rather than from a genuine increase in the occurrence of contaminated products.

RASFF notifications cannot be used to estimate dietary exposure, cancer incidence or disease burden without additional consumption and concentration data.

The reported country of origin should be interpreted as the country of origin recorded in the RASFF notification and does not necessarily correspond to the actual location where the product was manufactured.

## 4. Conclusions

The marked increase in acrylamide-related RASFF notifications following the 2017 and 2019 regulatory interventions most likely reflects the implementation of mandatory monitoring and mitigation measures throughout the food industry. However, the persistently high number of notifications recorded during 2023–2025 indicates that acrylamide remains a significant food safety challenge despite these regulatory efforts.

The NOVA-based analysis further demonstrated that ultra-processed foods constitute the principal category associated with high-risk acrylamide notifications, accounting for the vast majority of products classified as posing a serious risk and those subject to alert notifications. These findings identify ultra-processed foods as a priority target for future mitigation strategies and regulatory surveillance.

Given the widespread formation of acrylamide during both industrial food production and domestic cooking, dietary exposure is influenced by the combined effects of international food trade, manufacturing practices, and consumer cooking habits. Consequently, reducing population exposure requires an integrated approach encompassing optimized food processing technologies, strengthened regulatory oversight, continuous monitoring, and consumer education. The findings of this study provide further evidence to support the refinement of monitoring programs, the development of more effective mitigation strategies, and improved risk management of acrylamide throughout the food supply chain.

## Figures and Tables

**Table 1:** Acrylamide concentrations in major food groups reported in the most recent literature.

Food Type	Food Product	Acrylamide Concentration µg/kg (Average and Range)	Compliance with Benchmark Limits and Significant Notes	Country	Reference
Chips/crisps	Potato chips/crisps	3578 (nr)	Far exceeds EU benchmark (750 µg/kg).	Türkiye	[22]
		890 (160–2871)	51% of samples exceeded the EU benchmark.	Spain	[23]
		1116.6 (402–2103)	Average level exceeds the EU benchmark. MOEc mean value was 478, indicating a potential carcinogenic risk.	Italy	[24]
		565.2 (12–5310)	The most contaminated processed food in the study.	China	[25]
		630 (94–1905)	26% of samples exceeded the EU benchmark.	Spain	[26]
		178 (147–254)	Compliant; all samples below the EU benchmark.	Italy	
	Ready-to-eat chips, potato-based	177 (20–730)	No sample exceeded the EC benchmark.		[27]
	Ready-to-eat chips, corn-based	57 (4–155)	Significantly lower levels than potato-based chips.		
	Mixed vegetable chips	139 (nr)	Novel snack category.		
Breakfast Cereals	-	525.75 (160.08–1262.06)	Exceeds EU benchmark (300 µg/kg).	Lebanon	[28]
	-	573.02 (114.78–1246.8)	Exceeds EU benchmark. Higher levels in products from developing countries.	UAE	
	Wheat-based	202 (33–569)	Highest average among cereal types due to higher asparagine content.	Spain	[29]
	Puffed	284 (122–569)	Highest levels among processing types due to intense heat (200–300 °C).		
	Ready-to-eat	114 (<15–569)	95% of samples complied with EU benchmark levels.		
Biscuits	Buckwheat flour	78 (nr)	Lowest levels; stays below the benchmark even at high temperatures.	Spain	[30]
	Rye flour	1454 (nr)	Highest level among tested flours; significant increase with temperature.		
	Cornstarch	601 (411.31–900.16)	Exceeded EU benchmark. High levels due to invert sugar and leavening agents.	Brazil	[31]
	With apple and whole grains	1651 (nr)	Well above EU benchmark (350 µg/kg). Asparaginase reduced levels to 419 µg/kg.	Portugal	[32]
Crackers	-	169 (146–218)	Compliant; all samples below the EU benchmark of 400 µg/kg.	Italy	[26]
	-	96 (30–234)	Compliant; no sample exceeded the EU benchmark.	Spain	
Wafers	-	630 (197–831)	Highest average in category; 3/4 samples exceeded EC benchmark (350 µg/kg).	Brazil	[31]
	Cheese puff pastry	659. (nr)	The highest content in the cafeteria menu.	Türkiye	[33]
Popcorn	Microwave cooked	315 (85–529)	87% of samples exceeded the maize-based cereal benchmark (150 g/kg).	Spain	[34]
Sugar	Brown	438 (<5–5615)	Higher than fried potatoes in this study. Ginger brown sugar reached 272 µg/kg.	Taiwan	[35]
Coffee	Roasted powder	944.01 (326.60–944.01)	Exceeded EC benchmark of 450 µg/kg for roasted coffee, except for the Sidama variety.	Ethiopia	[36]
	-	194 (98–298)	Compliant; levels approx. 50% lower than the regulatory limit (400 µg/kg).	Spain	[26]
	-	228 (185–293)	Compliant; average levels below the EU regulatory limit of 400 µg/kg.	Italy	
Meals	Potato-based dishes (Cafeteria)	233 (nr)	Highest levels in the workplace cafeteria trial. French fries were below the 500 µg/kg limit.	Spain	[37]
	French fries (University Cafeteria)	369.6 (nr)	Compliant; below the EU reference value of 500 µg/kg.	Türkiye	[33]
	Baked chicken and potato	282.1 (nr)	Significant contributor to dietary exposure in cafeteria settings.		
	Sesame chicken	308.7 (nr)	High concentration detected in Turkish university cafeteria meals.		

nr—not reported.

## Data Availability

Data available in a publicly accessible repository (https://webgate.ec.europa.eu/rasff-window/screen/search (accessed on 20 January 2026)).

## Abbreviations

The following abbreviations are used in this manuscript:

ALARA	As low as reasonably achievable
IARC	International Agency for Research on Cancer
NFCs	Neoformed contaminants
RASFF	Rapid Alert System for Food and Feed

## References

[1] Vignesh A., Amal T.C., Vasanth K. (2024). Food contaminants: Impact of food processing, challenges and mitigation strategies for food security. Food Res. Int..

[2] Nerín C., Aznar M., Carrizo D. (2016). Food contamination during food process. Trends Food Sci. Technol..

[3] Taş N.G., Kocadağlı T., Gökmen V. (2022). Safety concerns of processed foods in terms of neo-formed contaminants and NOVA classification. Curr. Opin. Food Sci..

[4] Mogol B.A., Gökmen V. (2016). Thermal process contaminants: Acrylamide, chloropropanols and furan. Curr. Opin. Food Sci..

[5] Zahir A., Ge Z., Khan I.A. (2025). Public health risks associated with food process contaminants–A review. J. Food Prot..

[6] Adimas M.A., Abera B.D., Adimas Z.T., Woldemariam H.W., Delele M.A. (2024). Traditional food processing and Acrylamide formation: A review. Heliyon.

[7] Mirza Alizadeh A., Mohammadi M., Hashempour-baltork F., Hosseini H., Shahidi F. (2025). Process-induced toxicants in food: An overview on structures, formation pathways, sensory properties, safety and health implications. Food Prod. Process. Nutr..

[8] European Food Safety Authority (EFSA) (2015). Scientific Opinion on acrylamide in food. EFSA J..

[9] Benford D., Bignami M., Chipman J.K., Ramos Bordajandi L., European Food Safety Authority (EFSA) (2022). Assessment of the genotoxicity of acrylamide. EFSA J..

[10] International Agency for Research on Cancer List of Classifications. https://monographs.iarc.who.int/list-of-classifications/.

[11] European Commission (2017). Commission Regulation (EU) 2017/2158 of 20 November 2017 establishing mitigation measures and benchmark levels for the reduction of the presence of acrylamide in food. Off. J. Eur. Union.

[12] European Commission (2019). Commission Recommendation (EU) 2019/1888 of 7 November 2019 on the monitoring of the presence of acrylamide in certain foods. Off. J. Eur. Union.

[13] Nematollahi A., Meybodi N.M., Khaneghah A.M. (2021). An overview of the combination of emerging technologies with conventional methods to reduce acrylamide in different food products: Perspectives and future challenges. Food Control.

[14] Hee P.T.E., Liang Z., Zhang P., Fang Z. (2024). Formation mechanisms, detection methods and mitigation strategies of acrylamide, polycyclic aromatic hydrocarbons and heterocyclic amines in food products. Food Control.

[15] Louie J.C.Y. (2025). Are all ultra-processed foods bad? A critical review of the NOVA classification system. Proc. Nutr. Soc..

[16] European Commission RASFF Window. https://food.ec.europa.eu/food-safety/rasff_en.

[17] Microsoft Corporation (2026). Microsoft Excel.

[18] OpenStreetMap contributors (2026). OpenStreetMap Database.

[19] (2026). GeoNames. GeoNames Geographical Database. https://www.geonames.org.

[20] Žilić S., Sarić B., Mogol B.A., Kravić N., Hamzalıoğlu A., Simić M., Nikolić V., Gökmen V. (2024). Assessment of acrylamide content in corn-based snack products marketed in Serbia. J. Food Compos. Anal..

[21] Khaneghah A.M., Fakhri Y., Nematollahi A., Seilani F., Vasseghian Y. (2020). The Concentration of Acrylamide in Different Food Products: A Global Systematic Review, Meta-Analysis, and Meta-Regression. Food Rev. Int..

[22] Basaran B., Sadighara P. (2024). The level, human exposure, and health risk assessment of acrylamide in chips and breakfast cereals: A study from Türkiye. J. Food Compos. Anal..

[23] Mesías M., Martí S., Morales F.J. (2025). Monitoring Acrylamide in Potato Crisps in Spain using selected datasets from 2004-2025: Twenty Years of Trends, Current Status, and Compliance with European Regulation. Food Control.

[24] Cristaldi A., Pulvirenti E., Rapisarda P., Favara C., Castrogiovanni M., Conti G.O., Ferrante M. (2025). Determination of acrylamide levels in chips/crisps on the Italian market and exposure risk assessment. Food Chem. Toxicol..

[25] Liu Q., Pan F., Luo P., Zhou P. (2025). Levels of acrylamide in food products from a Chinese market and their risk assessment. J. Food Compos. Anal..

[26] Peris-Camarasa B., Pardo O., Nava V., di Bella G., Aleixandre C., Coscollà C. (2025). Presence of Acrylamide in key food sources within the European Mediterranean framework. Food Chem. Toxicol..

[27] Navarré A., Lombardi S., Paolillo A., Martínez-Alonso C., Rodríguez-Carrasco Y., Izzo L. (2025). Updated monitoring and risk assessment of acrylamide in traditional and emerging snack products in the Italian market. Food Control.

[28] Merhi A., Hassan H., Naous G.E.Z., Al Akoury C., Dimassi H., Serhan M., Alwan N., Taleb R.I. (2026). Dietary Exposure, Carcinogenic and Neurotoxic Risk Assessment of Acrylamide in Breakfast Cereals Marketed in Lebanon and the UAE. Appl. Food Res..

[29] Mesías M., García A., Morales F.J. (2025). Two decades of monitoring acrylamide in breakfast cereals: Impact of market reformulation and compliance with EU regulation. Food Chem. X.

[30] Roldán M., Mesias M., Morales F.J. (2026). Balancing nutrition and safety: Acrylamide and HMF in biscuits from different cereal flours. Food Chem. X.

[31] Rigo D., Gurak P.D., de Melo D.W., Garcia B.R., Scheid C., de Oliveira Merib J., de Souza Cruz F.M.T., Klein M.P. (2025). Acrylamide levels and health risk assessment from consumption of ultra-processed wheat flour-based products: A Brazilian perspective. J. Food Compos. Anal..

[32] Siopa J., Ribeiro M., Cosme F., Nunes F.M. (2025). Survey of acrylamide in marketed biscuits and industrial mitigation via dough composition and asparaginase. J. Food Compos. Anal..

[33] Kucuksullu E.K., Aytekin-Sahin G., Ozturk D. (2025). Hidden risks on the plate: Dietary acrylamide exposure in students using university cafeteria. J. Food Compos. Anal..

[34] Sebastià A., Fernández-Matarredona C., Castagnini J.M., Barba F.J., Berrada H., Moltó J.C., Pardo O., Esteve-Turrillas F.A., Ferrer E. (2025). Acrylamide content in popcorn from Spanish market: Risk assessment. Food Chem. Toxicol..

[35] Pan Y.A., Kang H.Y., Yeh A.I., Cheng W.C. (2026). Assessing Dietary Exposure to Acrylamide from Foods in Taiwan. J. Food Compos. Anal..

[36] Mengesha D., Retta N., Deribew H.A., Urugo M.M., Getachew P. (2025). Estimation of Dietary Acrylamide Exposure of Ethiopian Population Through Coffee Consumption. J. Food Prot..

[37] Delgado-Andrade C., González-Mulero L., Morales F.J., Mesías M. (2025). Acrylamide exposure in workplace cafeterias: Impact of consumer choices. Food Chem. Toxicol..

[38] Perestrelo S., Schwerbel K., Hessel-Pras S., Schäfer B., Kaminski M., Lindtner O., Sarvan I. (2024). Results of the BfR MEAL Study: Acrylamide in foods from the German market with highest levels in vegetable crisps. Food Chem. X.

[39] Nahid M., Bhuiyan M.N.I. (2025). Mitigating acrylamide formation in processed potato products: A comprehensive review of strategies, prevention, mitigation, and regulatory challenges. Food Chem. Adv..

[40] Kumari A., Bhattacharya B., Agarwal T., Paul V., Chakkaravarthi S. (2022). Integrated approach towards acrylamide reduction in potato-based snacks: A critical review. Food Res. Int..

[41] Hongyu Y., Hongling Y., Xiaolin L., Hongtao T., Siyue Y., Liyong L., Liang Z., Wei L. (2026). Exogenous additives for acrylamide mitigation in thermally processed foods: A review of safety risks and quality impacts. Food Chem..

[42] Navruz-Varlı S., Aksu S., Çergel E. (2025). What do we know about acrylamide levels in rice and rice products? A general overview. Food Chem. X.

[43] Törős G., Alibrahem W., Kharrat Helu N., Jevcsák S., Ferroudj A., Prokisch J. (2026). Acrylamide in Food: From Maillard Reaction to Public Health Concern. Toxics.

[44] Powers S.J., Mottram D.S., Curtis A., Halford N.G. (2021). Progress on reducing acrylamide levels in potato crisps in Europe, 2002 to 2019. Food Addit. Contam. Part A.

[45] Gielecińska I., Mojska H. (2023). Trends in the acrylamide content of potato products in Poland in the years 2004–2020. Food Control.

[46] Pesce F., Ponzo V., Mazzitelli D., Varetto P., Bo S., Saguy I.S. (2024). Strategies to reduce acrylamide formation during food processing focusing on cereals, children and toddler consumption: A review. Food Rev. Int..

[47] Strocchi G., Rubiolo P., Cordero C., Bicchi C., Liberto E. (2022). Acrylamide in coffee: What is known and what still needs to be explored. A review. Food Chem..

